# NMDAR Encephalitis Associated With Acute Chikungunya Virus Infection: A New Trigger?

**DOI:** 10.3389/fped.2020.00176

**Published:** 2020-04-30

**Authors:** Paulo Ribeiro Nóbrega, Norma Martins de Menezes Morais, Pedro Braga-Neto, Liziana Sofia da Silva Barros, Fernanda Paiva Pereira Honório, Alessandra Dellavance, Romana Hoftberger, Lívia Almeida Dutra

**Affiliations:** ^1^Division of Neurology, Department of Clinical Medicine, Universidade Federal do Ceará, Fortaleza, Brazil; ^2^Department of Pediatrics, Unichristus Medical School, Fortaleza, Brazil; ^3^Pediatric Service, Hospital Universitário Walter Cantídio, Universidade Federal Do Ceará, Fortaleza, Brazil; ^4^Center of Health Sciences, Universidade Estadual do Ceará, Fortaleza, Brazil; ^5^Laboratório Fleury, São Paulo, Brazil; ^6^Institute of Neurology, Medical University of Vienna, Vienna, Austria; ^7^Faculdade Israelita de Ciências da Saúde Albert Einstein, São Paulo, Brazil

**Keywords:** autoimmune, encephalitis, anti-NMDAR, Chikungunya, Arboviral diseases

## Abstract

**Background:** Anti-NMDAR encephalitis is the most frequent cause of autoimmune encephalitis. Chikungunya (CHIK) is an arbovirus responsible for outbreaks of fever, cutaneous rash and arthritis in underdeveloped countries, and a trigger for autoimmunity.

**Case Presentation:** We report a five-year-old male patient with fever, myalgia, headache and conjunctivitis for 5 days. After 1 week he developed tonic-clonic seizures and evolved with dystonia and oromandibular dyskinesia followed by onset of focal motor seizures, decreased level of consciousness, dysautonomia and central apnea. Brain MRI was normal, CSF analysis revealed 15 cells, protein 16.6 mg/dL and glucose 68 mg/dL. Anti-NMDAR antibodies were detected in serum and CSF after 3 weeks of symptom onset. CHIK serology was positive for both IgM and IgG, suggesting a recent infection. Dengue and Zika serologies were negative. CSF PCR for herpes viruses and arboviruses (CHIK, Dengue and Zika) were negative.

**Conclusion:** We report the occurrence of anti-NMDAR encephalitis after acute CHIK infection. The biphasic course, positivity for both CHIK IgM and IgG and negative CHIK CSF PCR results, as well as a dramatic response to immunotherapy suggest an immune-mediated pathogenesis. Because of the global epidemic of CHIK infection and unknown mechanisms involving CHIK and autoimmunity, patients with acute CHIK infections and neurological manifestations should be considered for antineuronal antibody testing.

## Introduction

Anti-NMDAR encephalitis is the most common form of autoimmune encephalitis and encompasses a wide range of clinical and paraclinical findings, including short-term memory deficit, decreased or altered level of consciousness, psychiatric symptoms, focal CNS findings or new onset seizures. The identification of these antibodies as biomarkers of treatable neurological syndromes has changed the approach to encephalitis and other inflammatory central nervous system (CNS) disorders (1). Chikungunya (CHIK) is an a arbovirus responsible for outbreaks of fever, cutaneous rash and arthritis in underdeveloped countries, and a trigger for autoimmunity (2–4). We report a patient that developed a typical presentation of anti-NMDAR encephalitis after an acute Chikungunya infection and discuss a possible causal relationship.

## Case Presentation

A five-year-old male non-Caucasian patient presented with fever, myalgia, headache, and conjunctivitis for 5 days. His past medical history was unremarkable, with normal psychomotor development, no family history neurological diseases and no consanguinity. The patient was born and lived in Ceará, northeast Brazil, and family reported no recent travels.

After 1 week he developed tonic-clonic seizures. Neurological examination was normal at this point. Complete blood count, liver functions and acute reactants were normal. Serologies for HSV-1, HSV-2, CMV, EBV, VZV, HIV, and toxoplasmosis were negative. Brain MRI was normal. Cerebrospinal fluid analysis revealed 15 cells, protein 16.6 mg/dL and glucose 68 mg/dL. He was started on acyclovir and ceftriaxone.

Two weeks after seizure onset, he presented with dystonia (Video 1) and oromandibular dyskinesia. On physical examination the patient was awake, his speech output was decreased, pupils were normal. Cranial nerves examination was unremarkable. Muscle strength was symmetric and deep tendon reflexes were normoactive and symmetric. One week later he developed focal motor seizures followed by decreased level of consciousness, dysautonomia, and central apnea. EEG showed extreme delta brush and valproate and phenytoin were started. He also received methylprednisolone followed by intravenous immunoglobulin with seizure resolution and improvement of level of consciousness, dysautonomia and orofacial dyskinesias within 2 weeks.

**Figure 1:**
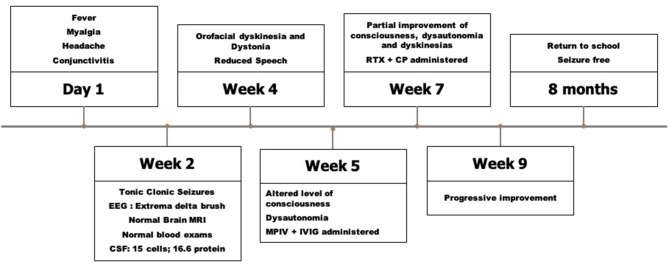
Timeline of clinical features, investigation and treatment of the case report. CSF, Cerebrospinal fluidl; MPIV, Intravenous methylprednisolone; MRI, Magnetic resonance imaging; IVIG, Intravenous immunoglobulin; RTX, Rituximab; CP, Cyclophosphamide.

Anti-NMDAR antibodies were detected in serum (titer 1:25600) and CSF (titer 1:1024) after 3 weeks of symptom onset using tissue and cell-based assays as previously reported (3). CHIK serology was positive for both IgM and IgG, suggesting a recent infection. Dengue and Zika serologies were negative. CSF PCR for herpes viruses and arboviruses (CHIK, Dengue and Zika) were negative. Whole body CT and testis ultrasound were normal. Because of partial improvement (persistence of orofacial dyskinesias and impaired speech), the patient received rituximab and cyclophosphamide with good response. After 8 months he is seizure-free and has returned to school with only mild restlessness and inattention. Figure 1 describes the timeline of clinical features, investigation and treatment of the case report.

## Discussion

We reported the occurrence of anti-NMDAR encephalitis after CHIK infection. The biphasic course, positivity for both CHIK IgM and IgG and negative CHIK CSF PCR results, as well as a dramatic response to immunotherapy suggest an immune-mediated pathogenesis. Differential diagnosis such as infectious encephalitis, such as Acute Demyelinating Encephalomyelitis (ADEM), Rasmussen and Bickerstaff encephalitis, central nervous system vasculitis, febrile infection related epilepsy syndrome (FIRES) and new-onset refractory status epileptics (NORSE) we ruled out.

The diagnostic approach to our case started with an acute febrile illness followed by tonic-clonic seizures. At this point the differential diagnosis was vast and included the infectious encephalitis related to herpes simplex family virus (HSV, CMV, EBV, VZV), measles and bacterial and fungal meningoencephalitis, which had to be ruled out by serology and CSF analysis. Empiric treatment for this agents was also initiated pending these results. The Chikungunya epidemic in our state, together with marked conjunctivitis, prompted a solicitation of CHIK serology. Other neurotropic viruses such as West Nile Virus and Saint Louis Encephalitis Virus are not endemic to our country.

Later, after typical orofacial dyskinesias were observed and a progression to altered level of consciousness, dysautonomia and central apnea, with the exclusion of infectious etiologies, a diagnosis of autoimmune encephalitis was proposed, and eventually confirmed by dosing of anti-NMDAR antibodies.

Anti-NMDAR is the most common autoimmune encephalitis, more frequently found among children and women (5). The occurrence of anti-NMDAR encephalitis following mycoplasma, influenza, VZV, HSV and EBV infections has been reported (3, 6). Release of antigens from infected and lysed neurons in the context of extensive inflammation, dysregulation of immunoregulatory pathways, and molecular mimicry are some of the mechanisms proposed for the link between infections and CNS autoimmunity (3).

Chikungunya is an arbovirus transmitted by Aedes vectors that causes a febrile illness usually accompanied by arthritis, cutaneous rash and conjunctivitis (4). It has become a global epidemic involving South America, Africa and Asia (7). Neurological complications occur 25% of the atypical presentations of the disease, in the first days and include encephalitis, myelitis, encephalomyelitis, optic neuritis, facial paralysis, sensorineural deafness, and acute flaccid paralysis such as Guillain–Barre syndrome (2, 4, 8). Similarly, to Zika, there are neonatal reports of chikungunya-associated encephalopathy (7) and evidence that CHIKV infects neurons and glial cells inducing apoptosis (9). Approximately 25–40% of patients develop immune mediated chronic arthropathy that includes musculoskeletal pain, arthralgia, or frank arthritis, lasting weeks or months after the acute attack. Immune-mediated mechanisms in CHIK infections are poorly understood, increased cytokine release, macrophage infection that may serve as a reservoir in some tissues, and further antigen presentation to lymphocytes (10, 11).

CHIK virus neurotropism has been demonstrated by intrathecal synthesis of chikungunya antibodies in acute infection complicated by encephalitis (12). CHIKV is associated with production of antiganglioside antibodies (8). Exposure of neuronal surface antigens after CHIK infection might be implicated in the development of anti-NMDAR antibodies, the same mechanism proposed for anti-NMDAR encephalitis after HSV-1 infection, an established neurotropic virus (13).

Preliminary data from our group hypothesized an increased incidence of autoimmune encephalitis in the period of 2014 to 2017 in Northeast Brazil, which might be related to an outbreak of arboviruses in the same period (14). Encephalitis associated with Chikungunya infection with good clinical response to immunoglobulin was also reported, raising the possibility of an immune-mediated mechanism for this presentation (15).

The main strength of the present report is to be the first to report an association of acute CHIK infection and the development of anti-NMDAR autoimmune encephalitis. Nonetheless, this is the report of a single case, which needs to be reinforced by similar observations. Although we have not detected CHIK PCR in the patient's CSF, this findings were reported in neurological manifestations associated with CHIK, including previously reported CHIK-encephalitis cases (15).

Because of the global epidemic of CHIK infection and unknown mechanisms involving CHIK and autoimmunity, patients with acute CHIK infections, and neurological manifestations should be considered for antineuronal antibody testing. This also raises concern that emergent and previously undocumented viruses could act as triggers for CNS autoimmunity.

## Ethics Statement

The studies involving human participants were reviewed and approved by Hospital Universitário Walter Cantídio. Written informed consent to participate in this study was provided by the participants' legal guardian/next of kin. Written informed consent was obtained from the individual(s), and minor(s)' legal guardian/next of kin, for the publication of any potentially identifiable images or data included in this article.

## Author Contributions

PN, PB-N, and LD: Conception and design of the work. PN, NM, LB, FH, AD, and RH: Acquisition, analysis, or interpretation of data for the work. PN, PB-N, LD, and RH: Drafting the work. All authors were involved in critical revision of the manuscript for important intellectual content.

## Conflict of Interest

The authors declare that the research was conducted in the absence of any commercial or financial relationships that could be construed as a potential conflict of interest.

## Supplementary Material

Video 1 Dystonia and oromandibular dyskinesia in a patient with anti-NMDAR encephalitis after an acute Chikungunya infection.
